# Analytical performance of Envisia: a genomic classifier for usual interstitial pneumonia

**DOI:** 10.1186/s12890-017-0485-4

**Published:** 2017-11-17

**Authors:** Yoonha Choi, Jiayi Lu, Zhanzhi Hu, Daniel G. Pankratz, Huimin Jiang, Manqiu Cao, Cristina Marchisano, Jennifer Huiras, Grazyna Fedorowicz, Mei G. Wong, Jessica R. Anderson, Edward Y. Tom, Joshua Babiarz, Urooj Imtiaz, Neil M. Barth, P. Sean Walsh, Giulia C. Kennedy, Jing Huang

**Affiliations:** Veracyte, Inc., 6000 Shoreline Ct., Suite 300, South San Francisco, 94080 California USA

**Keywords:** Envisia, Genomic classifier, Usual interstitial pneumonia, Transbronchial biopsy, Analytical verification

## Abstract

**Background:**

Clinical guidelines specify that diagnosis of interstitial pulmonary fibrosis (IPF) requires identification of usual interstitial pneumonia (UIP) pattern. While UIP can be identified by high resolution CT of the chest, the results are often inconclusive, making surgical lung biopsy necessary to reach a definitive diagnosis (Raghu et al., Am J Respir Crit Care Med 183(6):788–824, 2011). The Envisia genomic classifier differentiates UIP from non-UIP pathology in transbronchial biopsies (TBB), potentially allowing patients to avoid an invasive procedure (Brown et al., Am J Respir Crit Care Med 195:A6792, 2017). To ensure patient safety and efficacy, a laboratory developed test (LDT) must meet strict regulatory requirements for accuracy, reproducibility and robustness. The analytical characteristics of the Envisia test are assessed and reported here.

**Methods:**

The Envisia test utilizes total RNA extracted from TBB samples to perform Next Generation RNA Sequencing. The gene count data from 190 genes are then input to the Envisia genomic classifier, a machine learning algorithm, to output either a UIP or non-UIP classification result. We characterized the stability of RNA in TBBs during collection and shipment, and evaluated input RNA mass and proportions on the limit of detection of UIP. We evaluated potentially interfering substances such as blood and genomic DNA. Intra-run, inter-run, and inter-laboratory reproducibility of test results were also characterized.

**Results:**

RNA content within TBBs preserved in RNAprotect is stable for up to 14 days with no detectable change in RNA quality. The Envisia test is tolerant to variation in RNA input (5 to 30 ng), with no impact on classifier results. The Envisia test can tolerate dilution of non-UIP and UIP classification signals at the RNA level by up to 60% and 20%, respectively. Analytical specificity studies utilizing UIP and non-UIP samples mixed with genomic DNA (up to 30% relative input) demonstrated no impact to classifier results. The Envisia test tolerates up to 22% of blood contamination, well beyond the level observed in TBBs. The test is reproducible from RNA extraction through to Envisia test result (standard deviation of 0.20 for Envisia classification scores on > 7-unit scale).

**Conclusions:**

The Envisia test demonstrates the robust analytical performance required of an LDT. Envisia can be used to inform the diagnoses of patients with suspected IPF.

**Electronic supplementary material:**

The online version of this article (doi:10.1186/s12890-017-0485-4) contains supplementary material, which is available to authorized users.

## Background

Interstitial lung diseases (ILDs) are a group of diffuse parenchymal lung diseases characterized by varying patterns of inflammation and fibrosis, with an estimated prevalence in the US of 67–81 per 100,000 people [1]. ILDs can be difficult to accurately diagnose, show variable prognosis and have few good treatment options. When the cause of disease is unknown, differentiating among the possible ILDs is particularly important for providing an accurate prognosis and identifying the most effective treatment [1, 2]. A key characteristic of specific ILDs is the usual interstitial pneumonia (UIP) pattern, identifiable either by high-resolution computed tomography of the chest, or by pathology, typically obtained by an invasive surgical lung biopsy (SLB) procedure [3]. Unfortunately, not all ILD patients can tolerate surgery, and confident diagnoses of UIP from radiology or from pathology depends on expertise that may not be available in a local clinical setting. An accurate, broadly available test for UIP could inform the diagnosis of ILD without the need for surgery.

The Envisia genomic classifier is a laboratory developed test (LDT) which can detect, in minimally invasive transbronchial lung biopsies (TBB), a gene expression signature concordant with the UIP pathology pattern. The test uses a next-generation RNA-sequencing assay to enrich and amplify exonic transcripts from 15 ng total RNA, extracted and pooled from 3–5 TBBs per patient. Expression count data is input to a locked and validated machine learning algorithm for classification, developed for high rule-in performance characteristics, trained using a cohort of 90 ILD patients [4–6].

LDTs, which are regulated under the Center for Medicare and Medicaid Services’ CLIA laboratory regulations, undergo rigorous clinical and analytical evaluation as part of regulatory review [7]. An LDT must demonstrate accurate and reproducible performance under routine, day-to-day operation on the wide variety of patients and samples encountered in clinical practice. Substances reasonably or actually anticipated to contaminate patient specimens, either pre-analytically during sample collection or during the analytical testing process, must be evaluated for impact to test results and by extension, to patient reporting. In this study we evaluate the impact of potential pre-analytical interferents such as blood and adjacent lung tissue that may be inadvertently or variably sampled during bronchoscopic biopsy procedure. We also evaluate the impact of incomplete separation of DNA from RNA during laboratory processing, or errors in quantitation or pipetting of RNA. We assess the reproducibility of the test across multiple processing runs spanning several weeks within the CLIA-certified laboratory, in which operators, reagents, and equipment are varied. We also compare the accuracy of test results between two laboratories.

## Methods

### Specimens

Patients undergoing diagnostic evaluation for suspected ILD were enrolled, following informed consent, in a Western IRB-approved sample collection study (BRonchial sAmple collection for a noVel gEnomic test; BRAVE) intended to collect clinical samples for use in developing the molecular test. Transbronchial biopsy samples (two upper lobes and three lower lobes per patient, typically) were collected into RNAprotect preservative (QIAGEN, Valencia, CA) and stored on-site at 4 °C for up to 14 days. Specimens were shipped to Veracyte in a dedicated shipping container at a temperature of 4 °C, and frozen upon arrival at − 80 °C until extraction. Fresh frozen SLB specimens were also collected from BRAVE study subjects (e.g. BRAVE-1) undergoing this diagnostic procedure as part of their planned clinical evaluation. Fresh frozen surgical lung tissues with normal pathology, from patients with lung cancer (e.g. normal adjacent tissues), were also obtained commercially (Asterand USA). Blood samples were collected from healthy volunteers under a Liberty IRB-approved sample collection study and stored frozen in preservative. Pathology diagnoses were determined for BRAVE study subjects following a centralized review process, previously described [8]. Reference labels of UIP, non-UIP, or no label (non-diagnostic or unclassifiable fibrosis) were assigned based on a general categorization of the corresponding diagnostic pathology subtypes.

### RNA extraction, library preparation and RNA sequencing

The Envisia molecular test begins with the extraction of total RNA from TBB specimens. Total RNA was extracted using the AllPrep Micro Kit procedure (QIAGEN), with homogenization (TissueLyser and QIAshredder) up front of column-based nucleic acid isolation. RNA was quantitated using the QuantiFluor RNA System (Promega, Madison, WI), and samples assessed for RNA quality (measured as DV200, the percentage of RNA content in a sample > 200 nucleotides in length) and purity (evidence of contaminating DNA) using the Agilent RNA 6000 Pico assay (Agilent Technologies, Santa Clara, CA). A tissue lysate positive control material manufactured from frozen lung tissue and a negative control (lysis buffer) were used with pre-defined RNA yield and quality acceptance criteria to ensure the reproducibility of the extraction procedure. Individual TBB samples that met RNA yield QC criteria were combined in equal RNA mass proportion to form within-subject TBB pools for RNA sequencing. Sequencing libraries are generated using the TruSeq RNA Access Library Preparation kit using an automated liquid handling platform (Hamilton STAR, Reno, NV) and sequenced using the NextSeq system (Illumina, San Diego, CA). UIP and non-UIP total RNA controls were included in each sample batch starting from the library preparation. Pre-defined specifications for yield, quality, and Envisia classification of these control samples were used as batch acceptance criteria.

### Data analysis

To obtain an Envisia classifier result, expression counts for each gene (Additional file 1) were first normalized by the DESeq2 bioconductor package in R and transformed using variance stabilizing transformation (VST) [9]. The Envisia classifier score and a binary Envisia call, based on a locked score decision boundary, were determined using a locked machine learning algorithm (classifier) which uses expression count data from 190 genes as features [6].

TBB sample stability was evaluated using an ANOVA test of DV200 (RNA quality) over time. Linear mixed effect models were used to evaluate the effects of RNA input amount and genomic DNA interference. Splines were fitted to determine limits of detection for non-UIP and UIP gene expression signatures as well as tolerance to blood interference. A variable was claimed to be statistically significant if its *p*-value was less than 0.05. All 95% confidence intervals for standard deviations (SD) were obtained by bootstrap where the residuals of a linear mixed-effect model controlling for sample and other sources of variation (depending on the type of SD reported) were sampled with replacement. All data analysis was done in R version 3.2.3.

### *In silico* mixture modeling for analytical sensitivity

Mixtures of samples were modeled computationally (e.g., *in silico*) from the corresponding unmixed parent sample gene expression count data, and scored using the classifier. Technical replicates were used in all possible pairwise combinations, where available. A range of mixture proportions were simulated for each sample pair (one parent sample, one diluent sample), and the frequency of correct or expected classifier calls was determined, based on the UIP or non-UIP reference label assigned to the parent sample. Each simulation incorporated known technical variability for in vitro mixing and gene expression level measurements. The model was confirmed against mixtures for 8 patients performed in vitro (data not shown). We defined the simulated limit of detection as the most diluted mixture proportion where at least 90% of the simulated mixtures are called concordantly with the parent sample.

## Results

### Control materials

Multiple lots of UIP and non-UIP total RNA controls were manufactured using SLB tissue as source material. RNAs which reproducibly classified as UIP or non-UIP were selected for use as control materials in routine testing. All Envisia tests and studies outlined below included at least one UIP and one non-UIP total RNA control. All replicates of classification controls processed in this study classified as required.

### TBB specimen stability

**Fig. 1:**
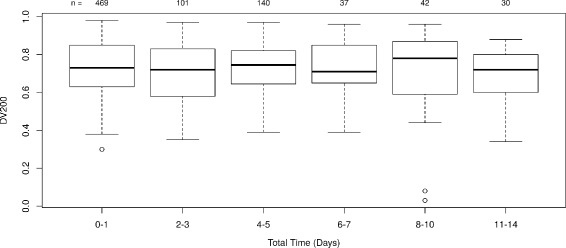
TBB specimen stability at 2-8 °C in RNAprotect. All samples from the BRAVE studies were analyzed at the time of RNA extraction. The resulting QC data were plotted as a function of the cumulative total storage time at 2-8 °C. The number of samples in each time window (n) for DV200 is shown at the top

To evaluate TBB sample stability under the collection and storage conditions specified for the Envisia test, we examined sample storage times at 2-8 °C (storage on-site prior to shipment and transit time during shipment) and frozen storage time at Veracyte for correlation to total RNA quality (DV200). No statistically significant difference in DV200 was observed among samples with a cumulative 2-8 °C storage time (on-site and in transit) of up to 14 days (*p*=0.21, Fig. 1), or among samples frozen at Veracyte for up to 100 days (*p*=0.85). As each sample is unique, the extent of sample preservation can vary from sample to sample, site to site, and subject to subject. While this inherent variability in sample quality could potentially mask or confound any storage time effects, we see no evidence among over 800 TBB samples of systemic storage effects on sample quality.

### Analytical sensitivity - total RNA input quantity

**Fig. 2:**
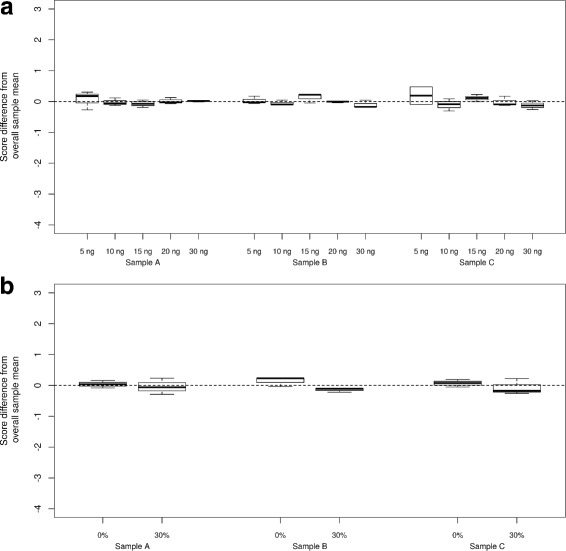
Analytical sensitivity and specificity of the Envisia test. The y-axis is a relative scale, with 0 representing the mean of each sample across all input levels (mean centered). Sample A and B are non-UIP samples. Sample C is a UIP sample. Each box represents test results from technical triplicates. Top (**a**) Effect of input mass variation on Envisia score. Bottom (**b**) Analytical specificity of the Envisia test against genomic DNA. The x-axis shows the percentage of total input mass, additional mass on top of the nominal 15ng total RNA, from genomic DNA

The Envisia test specifies that 15 ng of total RNA, representing a pool of RNA from 3 to 5 individual TBBs from each subject, is used as input to the library preparation procedure. Due to variation in the accuracy of quantitation (CV of technical replicates up to 20%, quantitation run-to-run CV up to 30%, data not shown) and pipetting (precision and accuracy 1% of volume typically), there is expected variation around the nominal total RNA input mass in routine practice. To characterize the tolerance of Envisia test results to variability in total RNA input, total RNA from one UIP and two non-UIP pooled TBB samples were processed in triplicate through the Envisia test at RNA input levels which vary substantially around the nominal input condition (5, 10, 15, 20, and 30 ng). Envisia test classification scores for each sample do not differ significantly as a function of RNA input, when evaluated with a linear mixed effect model (*p*-value =0.14) (Fig. 2 a). Although there is some evidence of higher variation of Envisia scores with low input amount of 5 ng, the Envisia test results are robust to 2 to 3-fold variation in RNA input, a range far in excess of the total technical variation anticipated under routine test conditions.

### Analytical sensitivity - limits of detection

The limit of detection for the Envisia test is the point where a typical UIP or non-UIP sample is diluted (with another tissue or RNA source) to the point where the sample no longer classifies in agreement with the reference pathology diagnosis. As the Envisia test is designed to be a high specificity (rule-in) test, we were particularly concerned with any interferent that could lead to a false UIP test result. We evaluated normal blood and tissues from multiple donors, and have found no examples of samples which classify as UIP (*n*=19, data not shown). Thus far, only samples with suspected or known UIP pathology classify as UIP. Therefore, the only currently plausible interferent of a non-UIP signature is a UIP sample. To determine the point of dilution where correct classification as non-UIP is lost, we first simulated mixtures of pooled non-UIP TBBs from four representative subjects with surgically-confirmed UIP lung biopsies from four additional subjects. This *in silico* simulation suggested that the non-UIP signal in non-UIP TBB pools can be classified correctly in dilutions that contain up to 59% [CI 57–60%] of a UIP diluent RNA by mass. We then selectively confirmed this predicted mixture proportion experimentally, using a TBB pool from one of the non-UIP subjects and a UIP surgical lung biopsy from a different subject. We tested three different proportions surrounding the estimated limit of detection: 55%, 65%, and 75% of UIP SLB diluent by total RNA mass (Table 1). When the RNA mixtures contain more than 65% of a UIP SLB sample (35% or less by mass of the non-UIP pooled TBB), this specific mixture tends to classify as UIP (Table 1).

**Table 1:** Envisia results from in vitro mixtures of non-UIP pooled TBB sample and UIP SLB sample

Sample	Non-UIP pooled TBB (%)	UIP SLB (%)	Envisia call
Non-UIP pooled TBB	100	0	Non-UIP
	45	55	Non-UIP
	35	65	UIP
	25	75	UIP
Pure UIP SLB	100	0	UIP

On the other hand, tolerance of the Envisia classifier to dilution of UIP signal was evaluated using in vitro total RNA mixtures derived from a TBB pool from one of the UIP subjects and an adjacent normal tissue from a different subject with lung cancer. Three different proportions were tested: 40%, 50%, and 60% of adjacent normal tissue diluent by total RNA mass (Table 2). The pure UIP TBB pool was called UIP by the Envisia classifier, whereas the pure adjacent normal tissue and all mixtures with it resulted in non-UIP Envisia calls (Table 2). A smoothed spline fitted to the in vitro sample scores suggests that the UIP signal in the UIP TBB pool can be classified correctly with up to 23% of adjacent normal tissue derived total RNA. A similar experiment was performed using a different UIP TBB pool mixed with a non-UIP SLB, with the smoothed spline suggesting that correct classification of the UIP TBB pool occurs in dilutions containing up to 18% of non-UIP RNA from the non-UIP SLB (data not shown).

**Table 2:** Envisia results from in vitro mixtures of UIP pooled TBB sample and adjacent normal tissue

Sample	UIP pooled TBB (%)	Adjacent normal tissue(%)	Envisia call
UIP pooled TBB	100	0	UIP
	60	40	Non-UIP
	50	50	Non-UIP
	40	60	Non-UIP
Adjacent normal tissue	0	100	Non-UIP

### Analytical specificity - blood

**Fig. 3:**
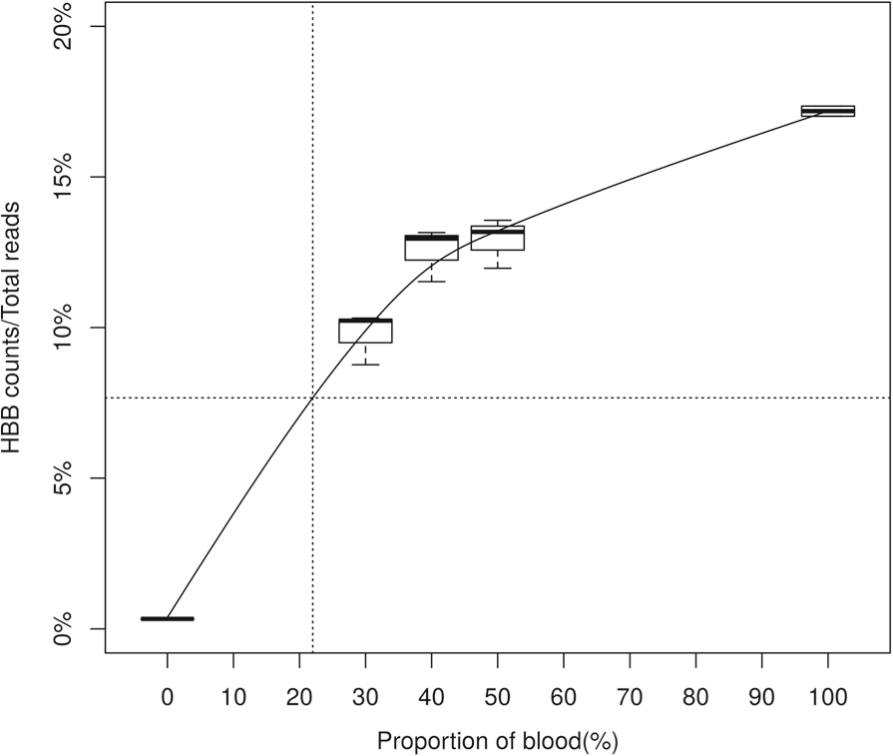
Proportion of HBB to total reads vs. proportion of blood contamination. Each boxplot represents the HBB count proportion for in vitro samples mixed with given proportion of blood. Vertical dashed line represents the level of blood (22%) that can cause a UIP sample to classify as non-UIP. Horizontal dashed line represents the proportion of HBB to total read counts for TBB samples mixed with 22% blood, which is 7%. The observed maximum value of blood content in TBB samples is on the order of < 1*%*

Visual inspection of samples collected during the BRAVE study showed that most of the samples have no visible blood contamination. However, it is possible that meaningful amounts of RNA from blood could be present in TBB samples. Blood contamination can be estimated using gene expression counts for blood-specific genes in clinical samples, specifically the proportion of hemoglobin beta / beta globin (HBB) gene counts to total read counts in TBB samples. The average proportion of HBB expression to total read counts is ∼ 0.3*%* for TBB samples (95th percentile is 1.3%), while the HBB read count proportion for pure blood samples is > 17*%* (Fig. 3), suggesting that measurable blood-specific gene expression in TBBs is, on average, 50-fold lower in TBBs than in pure blood.

To test the impact of blood on Envisia results, in vitro mixtures were created using RNA from one UIP TBB pool mixed with total RNA derived from a fresh whole blood sample, while maintaining the total RNA input to the test constant at 15 ng. Pure blood RNA, when processed unadulterated through the Envisia test, scores strongly as non-UIP (*n*=8 patients, not shown). Simulation of sample mixtures suggests that UIP signal is correctly detected a majority of the time (e.g., in 90% of simulated mixtures) in dilutions containing up to 27% [CI 26–28%] of normal blood RNA by mass. In vitro sample mixtures confirm that the UIP signal in a UIP TBB pool can be classified correctly with up to 22% of blood derived total RNA (not shown). For TBB samples mixed with 22% blood, we estimate the proportion of HBB gene counts to total reads is 7%, which far exceeds the upper limit of the proportion normally detected in TBBs. This suggests that blood contamination in excess of 20% has rarely occurred in TBBs collected for the development of the Envisia test.

### Analytical specificity - genomic DNA

Genomic DNA (gDNA) can rarely carry through the column-based RNA isolation procedure, contaminating the RNA tested by Envisia. The RNA QC process used in Envisia (Bioanalyzer) visually detects the presence of gDNA in TBB RNA down to levels of ∼ 3% of total nucleic acid mass (data not shown). However, in samples with specific RNA degradation patterns, the peak associated with gDNA in a Bioanalyzer trace could be partially or completely masked. Therefore, we tested contaminating gDNA as a potentially interfering substance. 15 ng of total RNA from one UIP and two non-UIP pooled TBB samples was spiked with gDNA (6.43 ng, or 30% of total nucleic acid mass) and processed in triplicate through the Envisia test. There was no significant difference in the Envisia score between samples with 30% gDNA and samples with no gDNA added (*p*-value =0.06) (Fig. 2 b).

### Assay reproducibility

Acceptable levels of run to run variation can be determined by evaluating the impact of additional noise to test sensitivity and specificity. We simulated the impact of increasing levels of random score variation *in silico* on Envisia classification scores for samples used to train the algorithm, and evaluated the impact to clinical sensitivity and specificity as estimated in cross validation. The simulation indicated that the Envisia classifier scores can tolerate total variation from all sources (reagents, operators, equipment, and run) of up to 7% of the score range (score SD ≤ 0.48 units on a ∼ 7-point scale), before a substantial reduction in sensitivity or specificity occurs. We then prospectively evaluated reproducibility of the Envisia test against this criterion, using pooled TBB samples from nine subjects covering the test score range and six control samples.

**Fig. 4:**
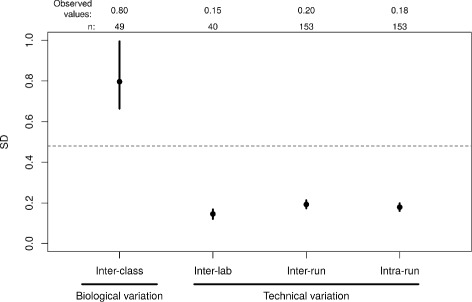
Comparison of Envisia score variability. The inter-class score SD includes biological variation between UIP and non-UIP samples and was computed from all samples passing quality control criteria from the BRAVE clinical studies. Dashed line: the maximum tolerable level of variation in Envisia scores derived from simulation. Black dots: observed values. Vertical lines: 95% CI. The number of data points used to calculate each SD (n) is shown at the top

Each sample was processed in at least triplicate across three experimental runs spanning several weeks in which reagent lots, operators, and equipment were varied. The within-run SD of Envisia scores on technical replicates of pooled TBB samples is 0.18 (95% CI 0.16 to 0.20; Fig 4), and the run-to-run SD of technical replicates is 0.20 (95% CI 0.17 to 0.21; Fig 4). Total variability in score ranges from 0.12 to 0.32 (*n*=153), well below the variability tolerance specification of 0.48. As a comparator, the total SD of scores between samples of different classes, e.g. between UIP and non-UIP samples, was measured to be 0.80 (95% CI 0.66-1.00; Fig 4).

### Inter-laboratory reproducibility

To assess whether the Envisia test performs equivalently in different laboratories, we processed pooled TBB samples from 20 different subjects in two different laboratories using different operators and equipment. The Envisia classifier calls for all samples were 100% concordant between the two laboratories, were highly correlated (*R* ^2^=0.99), and have an inter-laboratory pooled SD of scores of 0.15 (95% CI 0.12–0.17; Fig 4).

## Discussion

The Evaluation of Genomic Applications in Practice and Prevention (EGAPP) Working Group and the Centers for Disease Control’s ACCE Project (Analytic validity, Clinical validity, Clinical utility and associated Ethical, legal and social implications) have defined parameters which should be used to evaluate analytical validity of novel genomic tests [10, 11]. Here we report the results of recommended studies designed to test the analytical performance of the Envisia test.

We evaluated the entire process of sample collection, storage, shipping, sample processing, and classification for possible impact on the performance of the Envisia test. TBBs collected in the clinic according to the Envisia procedure are stable in cold storage, and total RNA extracted from them yields reproducible test results across a variety of conditions. The molecular assay was shown to be tolerant to routine RNA input quantity variation, and to possess limit of detection characteristics that provide reasonable robustness to sampling heterogeneity.

The limits of detection study indicated that it is very unlikely to convert a non-UIP sample into a UIP test result (e.g., a test false positive) since all currently known non-TBB sample types, such as normal blood and normal tissue, never score as UIP. This implies, but does not prove, that any apparent false positive result would be due to the sampling of actual UIP disease in the TBB. For this to be considered an actual false positive, the UIP disease must have been missed in the sample taken for pathology evaluation. Thus, sampling heterogeneity is the only known mechanism capable of generating an apparent false positive in the Envisia test.

On the other hand, our studies show that UIP samples can become mis-classified as non-UIP when mixed with normal adjacent tissue at > 20*%* proportion. This relatively modest resistance to the dilution of a true UIP sample is not surprising, as the decision boundary was deliberately designed to be resistant to false positives (e.g. to make the test highly specific). This has the natural tradeoff of a higher rate of false negatives, which is reflected in the lower resistance to dilution of UIP samples. Using gene expression data, we demonstrate that blood levels typically present in TBBs are far below the levels necessary to generate an Envisia false negative. Genomic DNA, another potential interferent introduced during sample processing in the laboratory, had no detectable impact to the Envisia scores. Taken together, these results suggest that the molecular biology underlying the Envisia test is robust to analytical as well as biological interference.

As outlined by EGAPP and ACCE, we used clinical samples with Envisia scores covering the entire range and concentrated around the decision boundary of the assay, and included positive and negative control materials and quality control and assurance measures [10]. The Envisia test gave identical results across multiple runs representing different reagent lots, operators, equipment, and laboratories, thus successfully meeting EGAPP level I internal validity criteria for reproducibility of test results [10]. The robustness of the Envisia test to induced variables, including those that may be encountered in clinical samples, supports that routine testing of TBB specimens is attainable at high confidence from the standpoint of analytical performance and reproducibility. The Envisia test is therefore suffciently analytically robust to be used in the routine clinical care of patients under evaluation for suspected ILD.

## Conclusions

In this study, we evaluated the analytical reproducibility and robustness of Envisia test results following the technical assessment criteria suggested by EGAPP and ACCE. We demonstrated that the Envisia test is analytically robust for use in the routine clinical care of patients under evaluation for suspected ILD.

## Additional file

Additional file 1 RNA-seq raw counts of samples used in Envisia analytical verification studies. This file includes the raw expression counts of 190 classifier genes across all samples (*n*=292) used in the analytical verification studies described in this manuscript; each row represents a gene and each column represents a sample. (CSV 199 kb)

## Abbreviations

ACCE: Analytic validity, Clinical validity, Clinical utility and associated Ethical, legal and social implications
CI: Confidence interval
CLIA: Clinical laboratory improvement amendments
CV: Coefficients of variation
EGAPP: Evaluation of Genomic applications in practice and prevention
IPF: Idiopathic pulmonary fibrosis
LDT: Laboratory developed test
SD: Standard deviation
SLB: Surgical lung biopsy
TBB: Transbronchial biopsy
UIP: Usual interstitial pneumonia
